# Accelerating into Immunization Agenda 2030 with momentum from China’s successful COVID-19 vaccination campaign during dynamic COVID Zero

**DOI:** 10.1186/s40249-023-01151-7

**Published:** 2023-10-16

**Authors:** Lance E. Rodewald

**Affiliations:** National Immunization Program, China CDC, Department of the National Immunization Program, Chinese Center for Disease Control and Prevention, 155 Changbai Road, Beijing, 102206 People’s Republic of China

**Keywords:** Vaccines, Immunization, Immunization program, Immunization Agenda 2030, COVID-19, Life course vaccination

## Abstract

China's immunization programs conducted a unified, tightly coordinated COVID-19 vaccination campaign during the dynamic COVID Zero period that reached well over 90% of the population with vaccines having > 90% effectiveness against serious-to-fatal COVID-19. The campaign was eight times the size of the annual routine national immunization program, administering 3.4 billion doses of vaccines while monitoring vaccine coverage, acceptability, safety, and effectiveness. Every asset of the routine immunization program had to be strengthened and expanded to attain high coverage and reach hundreds of millions of adults who had not been vaccinated since childhood. Program strengthening and expansion were in directions aligned with the World Health Organization's Immunization Agenda 2030, which has a vision that "everyone, everywhere, at every age fully benefits from vaccines for good health and well-being" and requires reaching all children, adolescents, and adults with lifesaving vaccines. Momentum from this campaign should not be lost but should be invested into achieving what is possible with a properly resourced national immunization program that is now proven to be capable of reaching everyone in the world’s largest country throughout the life course, and to do so with all vaccines recommended by the World Health Organization.

**Graphical Abstract:**

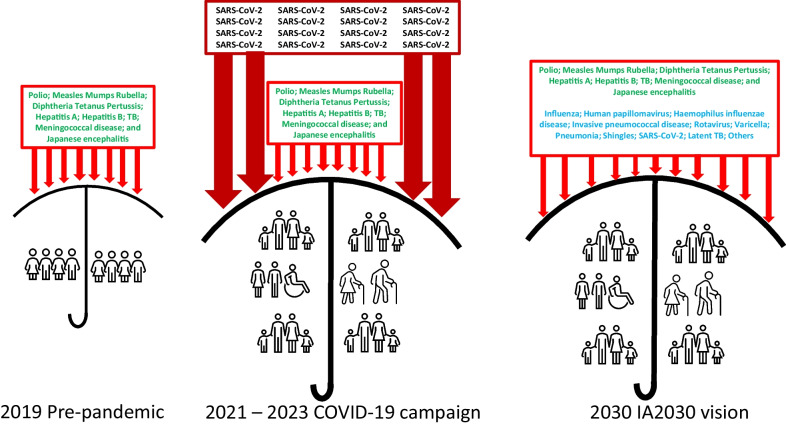

The job of the immunization program in China’s comprehensive COVID-19 response was to build population immunity with available vaccines to protect people from serious-to-fatal COVID-19 [1]. To do this job, China’s vertically integrated immunization program conducted an enormous COVID-19 vaccination campaign during the dynamic COVID Zero period while the risk of infection was kept very low, administering 3.4 billion doses of COVID-19 vaccine and reaching well over 90% of the all-ages population and over 95% of the elderly with China-produced vaccines that were > 90% effective against serious-to-fatal COVID-19 [2–4].

Based in part on Omicron’s characteristics and the vaccination campaign’s status, China adjusted the dynamic COVID Zero measures on 11 November 2022 and further adjusted remaining measures on 7 December 2022 with relaxation of testing, contact tracing, quarantine, travel, and other measures. Adjustments were followed by a large wave of infection in China’s mainland that swept through the country in less than 2 months, infecting the vast majority of people [2, 5]. Vaccine-induced immunity from the campaign greatly lowered the average disease severity during this huge epidemic wave, which ultimately led to the acquisition of near-population-wide hybrid immunity, synchronized by the short duration of the wave, and properly sequenced, with vaccination preceding infection—the safest way to acquire hybrid immunity from COVID-19.

## The campaign expanded the immunization program

The COVID-19 vaccination campaign was by far the largest expansion of China’s National Immunization Program since the inclusion of five new vaccines in 2008. With strong, multisectoral collaboration, the campaign extended the national program into new territory: new vaccines, new universal target populations, and new vaccine delivery platforms. Vaccination records were digitalized into Immunization Information Systems and health apps that helped manage everyone’s vaccinations and facilitated monitoring coverage, safety, and effectiveness of COVID-19 vaccines. Campaign vaccines were financed by the Medical Insurance Fund [6], establishing an innovative vaccination financing strategy that eliminated cost as a barrier to full vaccination by providing COVID-19 vaccines with no out-of-pocket expenses to individuals. Vaccination policy was updated to stay current with emerging evidence as new COVID-19 vaccines were approved and as SARS-CoV-2 evolved.

The COVID-19 campaign exercised and strengthened every asset of China’s immunization program—and in ways that are aligned with the World Health Organization’s (WHO) Immunization Agenda 2030 (IA2030). IA2030 is the thoughtfully developed global strategic plan for the current decade to use science, innovation, partnership, and country ownership to strengthen immunization programs so they can get the most out of vaccines for the populations they serve. IA2030 is designed to achieve the vision of a “world where everyone, everywhere, at every age fully benefits from vaccines for good health and well-being.”[7] In this Commentary, I’ll explore the alignment between IA2030 strategic priorities and China’s immunization program strengthening from the COVID-19 vaccination campaign, and argue for using momentum from the campaign to accelerate the immunization program into IA2030.

## Program expansion aligns with IA2030

IA2030 has seven strategic priorities: integration of immunization with primary care, country commitment to immunization fulfilling people-centered demand for vaccines, ensuring high and equitable coverage, vaccinating throughout the life course and integrated with essential services, management of outbreaks and emergencies, sustainable supplies of vaccines, and research and innovation. The COVID-19 vaccination campaign was well aligned with all seven of IA2030’s strategic priorities. The priorities that can impart the most momentum to the program and support the global IA2030 vision are ones that increase the number of vaccines in the routine program (Strategic Priority 2, Commitment and Demand) and broaden the age groups recommended for routine vaccination (Strategic Priority 4, Life Course & Integration). Another strategic priority with long-term benefit for China and the world is Strategic Priority 7, Research & Innovation. The COVID-19 campaign relied heavily on research and innovation, as exemplified by rapid and successful development of new and innovative COVID-19 vaccines, new vaccine delivery techniques, and new vaccination strategies in a fully digitalized immunization program that harnessed big data and artificial intelligence for monitoring and analyzing vaccine safety, effectiveness, and coverage.

IA2030 talks about “breadth of protection,” meaning implementing and sustaining high coverage with all WHO-recommended vaccines. The COVID-19 vaccination campaign conclusively proved that China’s immunization program can rapidly develop and introduce a new vaccine and achieve high vaccine coverage. The COVID-19 vaccination campaign was an astonishing eight times the size of the annual routine immunization program. This momentum and experience can be used to facilitate introduction of the vaccines that are recommended by WHO for all national programs but are not currently in China’s program. Recent [8, 9] and earlier [10] analyses of China’s national immunization program have recognized the disparity between the vaccines recommended by WHO and the vaccines included in China’s program and have recommended strategies to introduce new vaccines. A legislatively supported mechanism, the National Immunization Advisory Committee (NIAC), now exists that can recommend to government non-program vaccines that should be moved into the program based on evidence of preventable burden of disease and vaccine effectiveness, safety, cost effectiveness, and supply security [11]. Just as NIAC supported COVID-19 vaccination strategy, it can support evidence-based introduction of other vaccines. Moving human papillomavirus (HPV), pneumococcal conjugate (PCV), influenza, *Haemophilus influenzae* type b (Hib), varicella, and rotavirus vaccines into the national program would bring equitable and high coverage of these vaccines to well over 100 million young children and adolescents, preventing suffering from these infectious diseases while saving society money. Using domestically developed and produced vaccines will strengthen China’s vaccine industry and foster innovative development of new vaccines for use in China and for WHO prequalification and global use. For example, combining China’s Sabin-strain inactivated poliovirus vaccines into diphtheria, tetanus, acellular pertussis-, Hib-, and hepatitis B-containing combination vaccines could make “space” in the domestic routine immunization schedule for other vaccines while maintaining high polio vaccine coverage well into the future, for as long as is needed in China and elsewhere [12].

A thrust of IA2030 is life course vaccination. With its target population of everyone over the age of 3 years, China’s COVID-19 vaccination campaign exemplified life-course vaccination. COVID-19 vaccination was vigorously promoted to the elderly [13], people with comorbidities, health care workers and other working age adults, and school-age children. These are the same target populations for seasonal influenza vaccine. China CDC has recommended influenza vaccination of these populations for years [14], however uptake has been low except in several leading cities that have embraced influenza vaccination of these key target populations. The COVID-19 vaccination campaign proved that these populations can be reached to achieve high and equitable coverage. The COVID-19 vaccination experience can be used to make progress on influenza vaccination of these important target populations.

The maximum age for eligibility of National Immunization Program vaccines was recently raised from 14 to 18 years of age. This age range expansion can provide adolescents the opportunity to catch up on any program vaccinations they missed and to receive HPV vaccine once it is included in the program. But why stop at 18 years of age? The IA2030 vision is for the entire life course, as was the COVID-19 campaign. Program eligibility across all ages would enable immunization clinics, community health centers, and primary care providers to bring "everyone, everywhere, at all ages” the full benefits of vaccines. For adults, this could include not only influenza vaccine, but also pneumococcal and zoster vaccines in a comprehensive program integrated with primary care. Innovative financing of adult vaccines, as was done for COVID-19 vaccines with the Medical Insurance Fund, could support universal immunization program eligibility that would lead to equitable and high coverage for all—in good alignment with IA2030.

## China's immunization program can achieve the IA2030 vision

China’s National Immunization Program was strong before the COVID-19 pandemic—able to reach nearly every child with timely vaccination. Evidence for immunization program strength is the likely elimination of measles following a sustained, decades-long effort [15, 16]. But the COVID-19 vaccination campaign demonstrated resolutely that when adequately resourced and prioritized, the immunization program can do much more. In physics, momentum has direction, and the direction of the COVID-19 vaccination campaign’s momentum was in exact alignment with IA2030 strategic priorities. Let us use that campaign momentum and bring together immunization stakeholders and policy makers to achieve the IA2030 vision with China’s National Immunization Program so that “everyone, at every age, fully benefits from vaccines for good health and well-being.”

## Data Availability

All data included in the commentary are from cited references and are publicly available.

## Abbreviations

Hib: Haemophilus influenzae type b
IA2030: Immunization Agenda 2030
HPV: Human papillomavirus
NIAC: National Immunization Advisory Committee
PCV: Pneumococcal conjugate
WHO: World Health Organization

## References

[1] An ZJ, Wang FZ, An P (2021). Vaccination strategy and challenges for consolidating successful containment of covid-19 with population immunity in China. BMJ.

[2] China CDC. COVID-19 Clinical and surveillance data — December 9, 2022 to April 27, 2023, China. China CDC Weekly under COVID-19 section. https://weekly.chinacdc.cn/. Accessed 10 July 2023.

[3] Rodewald LE, Wu D, Yin ZD, Feng ZJ (2022). Vaccinate with confidence and finish strong. China CDC Weekly.

[4] Huang ZY, Xu SF, Liu JC (2022). Effectiveness of inactivated and Ad5-nCoV COVID-19 vaccines against SARS-CoV-2 Omicron BA. 2 variant infection, severe illness, and death. BMC Med.

[5] Information Office, Henan Provincial Government. Henan has passed the peak of the epidemic and the infection rate of the SARS-CoV-2 is 89.0%. https://www.henan.gov.cn/2023/01-09/2669240.html. Accessed 10 July 2023.

[6] http://www.gov.cn/xinwen/2022-04/03/content_5683325.html. Accessed 18 May 2023.

[7] O’Brien KL, Lemango E, Nandy R, Lindstrand A (2022). The immunization Agenda 2030: a vision of global impact, reaching all, grounded in the realities of a changing world. Vaccine.

[8] Chen S, Yao L, Wang WB, Tang SL (2022). Developing an effective and sustainable national immunisation programme in China: issues and challenges. Lancet Public Health.

[9] Dai PX, Wang Q, Jia MM, Leng ZW, Xie SY, Feng LZ, Yang WZ (2023). Driving more WHO-recommended vaccines in the National Immunization Program: issues and challenges in China. Hum Vaccin Immunother.

[10] Lei ZL, Li QL, Li L (2014). Review the significant achievement in EPI with full awareness of the challenges to implement EPI with full confidence in the future. Chin J Vaccin Immunization.

[11] Feng ZJ (2019). Vaccine policy as evidence-based public health decision making in action. China CDC Weekly.

[12] Polio Post-Certification Strategy: a risk mitigation strategy for a polio-free world. Geneva: World Health Organization; 2018. Licence: CC BY-NC-SA 3.0 IGO. https://polioeradication.org/wp-content/uploads/2018/04/polio-post-certification-strategy-20180424-2.pdf. Accessed 13 Oct 2023.

[13] Zang SJ, Zhang X, Qu ZQ, Chen X, Hou ZY (2022). Promote COVID-19 vaccination for older adults in China. China CDC Weekly.

[14] National Immunization Advisory Committee (NIAC) Technical Working Group on Influenza Vaccination (2022). Technical guidelines for seasonal influenza vaccination in China (2022–2023). Chin J Epidemiol.

[15] Durrheim DN, Xu AQ, Baker MG, Hsu LY, Takashima Y (2023). China has the momentum to eliminate measles. Lancet Reg Health West Pac.

[16] Song QW, Ma C, Hao LX, Wang FZ, An ZJ, Yin ZD, Wang HQ (2023). Effects of three major immunization interventions on measles control—China, 1952–2021. China CDC Weekly.

